# *QuickStats:* Percentage* of Currently Employed Adults Aged 18–64 Years Who Have Paid Sick Leave,^†^ by Poverty Status^§^ — National Health Interview Survey,^¶^ United States, 2008 and 2018

**DOI:** 10.15585/mmwr.mm6846a6

**Published:** 2019-11-22

**Figure:**
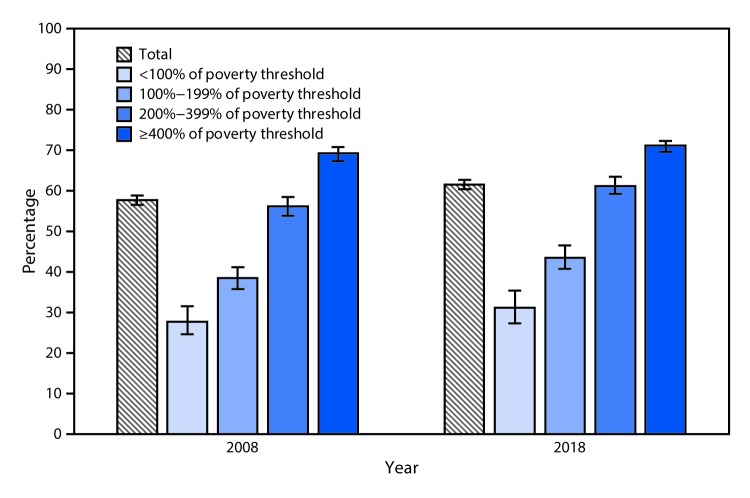
The percentage of currently employed adults aged 18–64 years who have paid sick leave increased from 57.8% in 2008 to 61.7% in 2018. For both 2008 and 2018, the percentage of employees with paid sick leave increased with family income. In 2018, the percentage with paid sick leave was 31.5% for those with incomes <100% of the poverty threshold, increasing to 71.4% for those with incomes ≥400% of the poverty threshold. The percentage of employees with paid sick leave increased from 2008 to 2018 in all poverty groups, although the increase was not significant for those with incomes <100% of the poverty threshold or for those with incomes ≥400% of the poverty threshold.

**Source:** National Health Interview Survey, 2008 and 2018. https://www.cdc.gov/nchs/nhis/index.htm.

